# Comprehensive Analysis of the Effect of Maternal and Paternal Demographics and Lifestyle Factors on the Embryo Quality and In Vitro Fertilization Outcomes: A Retrospective Study in North Eastern India

**DOI:** 10.7759/cureus.35546

**Published:** 2023-02-27

**Authors:** Sangeeta Kumari, Kalpana Singh, Bhawana Tiwary, Shubhanti Kumari, Huma Nishat

**Affiliations:** 1 Reproductive Medicine, Indira Gandhi Institute of Medical Sciences, Patna, IND

**Keywords:** maternal demographics, paternal demographics, ivf, embryo quality, lifestyle habits

## Abstract

Background

Lifestyle habits and demographic characteristics are strongly associated with sperm and oocyte quality and are important co-variates in fertility. However, their effect on the pre-implantation embryo quality in *in vitro* fertilization (IVF) has not been explored widely. The present retrospective study aimed to explore the effect of maternal and paternal demographic and lifestyle factors on the pre-implantation embryo quality in IVF.

Methodology

Women in the age group of 21 to 40 years undergoing IVF (n=105) in the Department of Reproductive Medicine, Indira Gandhi Institute of Medical Sciences, Patna, Bihar, and their partners were recruited in the study. Maternal and paternal charts were reviewed, and the demographic, lifestyle habit related data, and data related to oocyte retrieval, oocyte quality, and embryo quality were retrieved in a predesigned spreadsheet. Appropriate statistical analysis was conducted using SPSS Version 21 to evaluate the association of the studied maternal and paternal factors with oocyte and embryo quality. P-values less than 0.05 were considered to be significant.

Results

Maternal factors such as tubal blockage (p=0.02) and residence in an industrial locality (p=0.001) were found to be significantly associated with the quality of oocytes. None of the maternal factors studied were associated with embryo quality; however, day 3 and day 5 embryo quality was significantly associated with educational status of the male partners (p=0.02), smoking (p=0.05), and chewing tobacco (p=0.01). Day 5 embryo quality was also associated with residence in an industrial locality of the male partners (p=0.04).

Conclusions

Paternal lifestyle habits such as smoking, chewing tobacco, and demographic characteristics such as education and proximity to an industrial area were all related to poor embryo quality. Maternal factors such as tubal blockage and residence of industrial locality were found to be significantly associated with the quality of oocytes.

## Introduction

Infertility is a disorder that is influenced by a number of intricate physiological, anatomical, and genetic factors. Increasing body of research shows that infertility is frequently associated with demographic and lifestyle characteristics such as age, body mass index (BMI), occupation, level of education, food preferences, smoking, and alcohol intake. There is a lot of evidence that lifestyle factors that are modifiable, including smoking and body weight, have a negative effect on in vitro fertilization (IVF), which may alter how effectively IVF works [1,2]. BMI and smoking behavior are both related to a higher risk of IVF failure [3-8]. Alcohol intake before the start of assisted reproductive technology (ART) had no impact on the outcomes, despite being a key factor negatively associated with ART outcomes such as fertilization, embryo quality, and implantation [9].

The relationship between exercise and the outcomes of pregnancies is controversial. Morris et al. reported that women who exercised more than 4 hours per week had a lower chance of having a live birth [10]. However, a different study found that people who had greater active lifestyle and exercise/sports indices over the previous year were more likely to have a clinical pregnancy [11].

These lifestyle habits have been reported to equally affect fertility in men. According to reports, these lifestyle choices have an equal impact on men's fertility. Alcohol intake and smoking have been shown to have a deleterious impact on semen quality [12,13]. Both males’ and females' reproductive status is influenced by demographic factors and lifestyle choices made by parents. These factors are associated with the success of ART. Since the majority of these factors are modifiable, they should be thoroughly examined in couples considering or having IVF. The present study aimed to comprehensively analyze the maternal and paternal demographic and lifestyle parameters and to evaluate their association with oocyte and embryo quality during IVF.

## Materials and methods

This is a retrospective study conducted in the Department of Reproductive Medicine, Indira Gandhi Institute of Medical Sciences (IGIMS), Patna, Bihar. Women in the age group of 21 to 40 years undergoing IVF in the Department of Reproductive Medicine, IGIMS, and their partners were recruited in the study. Patients not falling into the specified age category and those with incomplete data were excluded from the study.

Ethics statement

Ethical clearance was obtained from the Institutional Ethics Committee (IEC), IGIMS, Patna, Bihar, before initiating the study (35/IEC/IGIMS/2021).

Retrospective data collection

Data were collected from February 2019 to December 2022. Maternal and paternal charts were reviewed, and the demographic data, lifestyle habit related data, and data related to the IVF procedure and outcomes such as type of IVF cycle, oocyte retrieval, fertilization, number of viable embryos, embryo quality, embryo transfer, implantation success, and pregnancy rate in IVF cycle were retrieved in a predesigned spreadsheet.

Oocyte quality assessment

Quality of oocytes was assessed on the basis of morphological features (cytoplasmic content, perivitelline space, first polar body, zona pellucida, and shape). Oocytes were categorized as follows: good (proper cytoplasmic content and perivitelline space without granulation, appearance of first polar body without fragmentation, proper thickness of zone pellucida, absence of vacuoles, refractive bodies, dark incorporation, fragmentation, lipid droplets, dense granules, and spots), moderate (less than 25% fragmentation), and poor (irregular shape and perivitelline space, presence of granules and vacuoles, more than 50% fragmentation, and fragmented polar body).

Embryo quality assessment

Day 3 embryos were assessed for quality on the basis of morphological criteria (blastomere number, blastomere regularity, nuclear content, and fragmentation rate). The following grading system was used: good (equally sized symmetrical blastomeres without fragmentation), moderate (unevenly sized blastomeres without fragmentation), and poor (embryos with 50% or more fragmentation).

Day 5 embryos were assessed for quality on the basis of morphological criteria such as inner cell mass formation, trophectoderm layer formation, blastocoel cavity formation, and fragmentation. The following grading system used followed:

· Grade A (good): numerous and tightly packed inner cell mass; trophectoderm is many-celled and organized in the epithelium

· Grade B (moderate): several and loosely packed inner cell mass; trophectoderm is several-celled and organized in the loose epithelium

· Grade C (poor): few and unorganized inner cell mass and trophectoderm

Statistical analysis

Statistical analysis was performed using SPSS software Version 21 (IBM Corp., Armonk, NY). Normality of the data was checked using the Shapiro-Wilk test. Continuous variables were represented as mean±SD and categorical variables were expressed as frequency (%). Student’s t-test and chi-square tests were used to compare continuous and categorical variables between groups. Univariate and multivariate logistic regression analyses were performed to evaluate the correlation/association of the paternal and maternal demographic and lifestyle parameters with embryo quality, implantation rates, and IVF outcome/pregnancy rate. P-values less than 0.05 were considered to be significant.

## Results

Baseline characteristics of the participants

The study included 105 females and their partners. The baseline characteristics of the females and the male partners are described in Tables 1, 2, respectively.

**Table 1:** Baseline characteristics of the female partners

	N	Minimum	Maximum	Mean	Std. Deviation
Age (years)	105	21.0	40.0	31.057	4.1944
Weight (kg)	105	40.0	89.0	57.219	8.9829
Height (feet)	105	4.1	6.0	5.230	0.3095
BMI (kg/m^2^)	105	13.5	35.1	22.673	4.0776
Duration of marriage (years)	105	2.0	19.0	9.248	4.1319
No of oocytes retrieved	105	2.0	11.0	5.495	2.5082

**Table 2:** Baseline characteristics of the male partners

	Minimum	Maximum	Mean	Std. Deviation
Age (years)	21.0	49.0	36.543	5.7530
Weight (kg)	55.0	82.0	68.105	4.8515
Height (feet)	5.0	7.0	5.857	0.2974
BMI (kg/m^2^)	14.9	26.2	21.514	2.4957
Sperm count (million/mL)	2.0	250.0	63.248	49.6195
Motility (%)	0.0	90.0	45.190	19.7416

Maternal demographic and lifestyle factors

The mean age of the females was 31.06±4.19 years. The mean weight and height were 57.22±8.98 kg and 5.23±0.31 feet, respectively. The mean BMI was 22.67±4.08 kg/m^2^. The mean duration of marriage was 9.25±4.13 years (Table 1).

None of the females had a history of consanguinity, 70 (66.7%) females had primary infertility, 35 (33.3%) had a history of previous conception, none of them used contraceptives, 35 (31.3%) females had tubal blockage, only two (1.8%) females had an abnormal menstrual history, nine (8%) were taking regular medications for conditions such as hypothyroidism and hypertension, and none of them were consuming alcohol, smoking, or chewing tobacco. Only eight (7.1%) females were doing regular exercise/yoga. Nineteen (17%) females were residing in an industrial locality and none of the participants lived in a high-altitude area, 89 (79.5%) of the females were drinking tea/coffee, and 97 (86.6%) of the females were home-makers.

Paternal demographic and lifestyle factors

The mean age of the males was 36.54±5.75 years. The mean weight and height were 68.11±4.85 kg and 5.86±0.31 feet, respectively. The mean BMI was 21.51±2.51 kg/m^2^. The mean sperm count was 63.25±49.62 million/mL. The mean sperm motility % was 45.19±9.74 (Table 2).

None of the male partners had a history of consanguinity, three (2.7%) had a history of surgery, 21 (18.8%) were on regular medication for conditions such as hypertension, six (5.4%) were doing regular exercise/yoga, 18 (16.1%) were living in an industrial locality, none of them lived in a high-altitude area ever, 102 (91.1%) were drinking tea/coffee regularly, 34 (30.4%) were smoking, 25 (22.3%) were consuming alcohol, and 16 (14.3%) chewed tobacco.

Oocyte quality and maternal factors

Demographic and clinical characteristics were assessed for any association with quality of oocytes. Among the maternal factors, tubal blockage (p=0.02) and residence in an industrial locality (p=0.001) were found to be significantly associated with the poor quality of oocytes (Table 3). No other maternal factors were significantly associated with oocyte quality.

**Table 3:** Oocyte quality and maternal factors Chi-square test was conducted to assess the association of different maternal factors with oocyte quality. P-values less than 0.05 were considered to be significant.

	Good (n=45)	Moderate (n=30)	Poor (n=30)	p-value
Education				
No school	30 (66.7)	18 (60.0)	18 (60.0)	0.89
Primary	1 (2.2)	0	0	
High school	10 (22.2)	8 (26.7)	9 (30.0)	
Intermediate	4 (8.9)	4 (13.3)	3 (10.0)	
Parental consanguinity	0	0	0	NA
Type of infertility				
Primary	25 (55.6)	23 (76.7)	22 (73.3)	0.11
Secondary	20 (44.4)	7 (23.3)	8 (26.7)	
Contraceptive used	0	0	0	NA
Menstrual history (abnormal)	0	0	2 (6.7)	0.08
Tubal blockage	15 (33.3)	5 (16.7)	15 (50.0)	0.02
Medication on a regular basis	7 (15.6)	0	2 (6.7)	0.06
Exercise/yoga/meditation	7 (15.6)	1 (3.3)	2 (6.7)	0.06
Industrial locality	3 (6.7)	4 (13.3)	12 (40)	0.001
Tea/coffee	41 (91.1)	24 (80.0)	24 (80.0)	0.29
Smoking	0	0	0	NA
Alcohol	0	0	0	NA
Tobacco	0	0	0	NA
BMI				
Underweight (<18.5 kg/m^2^)	8 (17.8)	1 (3.3)	5 (16.7)	0.27
Normal weight (18.5-24.9 kg/m^2^)	25 (55.6)	25 (83.3)	17 (56.7)	
Overweight (25.0-29.9 kg/m^2^)	8 (17.8)	3 (10.0)	6 (20.0)	
Obese (>30 kg/m^2^)	4 (8.9)	1 (3.3)	2 (6.7)	

Embryo quality and maternal factors

None of the studied maternal factors were found to be associated with day 3 and day 5 embryo quality (Tables 4, 5).

**Table 4:** Day 3 embryo quality and maternal factors Chi-square test was conducted to assess the association of different maternal factors with day 3 embryo quality. P-values less than 0.05 were considered to be significant.

	Good (n=57)	Moderate (n=17)	Poor (n=31)	p-value
Education				
No school	32 (56.1)	14 (82.4)	20 (64.5)	0.11
Primary	0	0	1 (3.2)	
High school	15 (26.3)	3 (17.6)	9 (29.0)	
Intermediate	10 (17.5)	0	1 (3.2)	
Parental consanguinity	0	0	0	NA
Type of infertility				
Primary	35 (61.4)	11 (64.7)	24 (77.4)	0.31
Secondary	22 (38.6)	6 (35.3)	7 (22.6)	
Contraceptive used	0	0	0	NA
Menstrual history (regular)	1 (1.8)	1 (5.9)	0	0.36
Tubal blockage	16 (28.1)	8 (47.1)	11 (35.5)	0.33
Medication on a regular basis	4 (7.0)	3 (17.6)	2 (6.5)	0.34
Exercise/yoga/meditation	7 (12.3)	1 (5.9)	2 (6.5)	0.85
Industrial locality	12 (21.1)	4 (23.5)	3 (9.7)	0.34
Tea/coffee	47 (82.5)	13 (76.5)	29 (93.5)	0.22
Smoking	0	0	0	NA
Alcohol	0	0	0	NA
Tobacco	0	0	0	NA
BMI				
Underweight (<18.5 kg/m^2^)	8 (14.0)	4 (23.5)	2 (6.5)	0.23
Normal weight (18.5-24.9 kg/m^2^)	39 (68.4)	8 (47.1)	20 (64.5)	
Overweight (25.0-29.9 kg/m^2^)	6 (10.5)	5 (29.4)	6 (19.4)	
Obese (>30 kg/m^2^)	4 (7.0)	0	3 (9.7)	

**Table 5:** Day 5 embryo quality and maternal factors Chi-square test was conducted to assess the association of different maternal factors with day 5 embryo quality. P-values less than 0.05 were considered to be significant.

	Good (n=40)	Moderate (n=32)	Poor (n=33)	p-value
Education				
No school	23 (57.5)	22 (68.8)	21 (63.6)	0.19
Primary	0	0	1 (3.0)	
High school	9 (22.5)	8 (25.0)	10 (30.3)	
Intermediate	8 (20.0)	2 (6.3)	1 (3.0)	
Graduate	0	0	0	
Parental consanguinity	0	0	0	NA
Type of infertility				
Primary	22 (55.0)	23 (71.9)	25 (75.8)	0.13
Secondary	18 (45.0)	9 (28.1)	8 (24.2)	
Contraceptive used	0	0	0	NA
Menstrual history	40 (100.0)	30 (93.8)	33 (100.0)	0.11
Tubal blockage	12 (30.0)	13 (40.6)	10 (30.3)	0.58
Medication on a regular basis	3 (7.5)	4 (12.5)	2 (6.1)	0.62
Exercise/yoga/meditation	6 (15.0)	2 (6.3)	2 (6.1)	0.06
Industrial locality	7 (17.5)	9 (28.1)	3 (9.1)	0.14
Tea/coffee	32 (80.0)	26 (81.3)	31 (93.9)	0.21
Smoking	0	0	0	NA
Alcohol	0	0	0	NA
Tobacco	0	0	0	NA
BMI				
Underweight (<18.5 kg/m^2^)	3 (7.5)	9 (28.1)	2 (6.1)	0.16
Normal weight (18.5-24.9 kg/m^2^)	27 (67.5)	18 (56.3)	22 (66.7)	
Overweight (25.0-29.9 kg/m^2^)	7 (17.5)	4 (12.5)	6 (18.2)	
Obese (>30 kg/m^2^)	3 (7.5)	1 (3.1)	3 (9.1)	

Embryo quality and paternal factors

Day 3 embryo quality was observed to be positively associated with educational status of the male partners (p=0.02) and negatively associated with smoking (p=0.05) and chewing tobacco (p=0.01). Day 5 embryo quality was also found to be positively associated with the educational status of the male partners (p=0.05) and negatively associated with smoking (p=0.01), chewing tobacco (p=0.01), and residence in an industrial locality (p=0.04) (Tables 6, 7).

**Table 6:** Day 3 embryo quality and paternal factors Chi-square test was conducted to assess the association of different paternal factors with day 3 embryo quality. P-values less than 0.05 were considered to be significant.

	Good (n=57)	Moderate (17)	Poor (31)	p-value
Education				
No school	16 (28.1)	6 (35.3)	1 (3.2)	0.02
Primary	6 (10.5)	2 (11.8)	6 (19.4)	
High school	10 (17.5)	4 (23.5)	5 (16.1)	
Intermediate	5 (8.8)	4 (23.5)	9 (29.0)	
Graduate	20 (35.1)	1 (5.9)	10 (32.3)	
Parental consanguinity	0	0	0	NA
Surgical history	2 (35.0)	0	1 (3.2)	0.74
Medication on a regular basis	14 (24.6)	2 (11.8)	5 (16.1)	0.42
Exercise/yoga/meditation	3 (5.3)	2 (11.8)	1 (3.2)	0.47
Industrial locality	10 (17.5)	5 (29.4)	3 (9.7)	0.22
Tea/coffee	55 (96.5)	16 (94.1)	31 (100)	0.46
Smoking	13 (22.8)	6 (35.3)	15 (48.4)	0.05
Alcohol	11 (19.3)	3 (17.6)	11 (35.5)	0.19
Tobacco	4 (7.0)	6 (35.3)	6 (19.4)	0.01
BMI				
Underweight (<18.5 kg/m^2^)	11 (19.3)	0	2 (6.5)	0.10
Normal weight (18.5-24.9 kg/m^2^)	40 (70.2)	14 (82.4)	24 (77.4)	
Overweight (25.0-29.9 kg/m^2^)	3 (5.3)	3 (17.6)	3 (9.7)	
Obese (>30 kg/m^2^)	3 (5.3)	0	2 (6.5)	

**Table 7:** Day 5 embryo quality and paternal factors Chi-square test was conducted to assess the association of different paternal factors with day 5 embryo quality. P-values less than 0.05 were considered to be significant.

	Good (n=40)	Moderate (n=32)	Poor (n=33)	p-value
Education				
No school	12 (30.0)	10 (31.3)	1 (3.0)	0.05
Primary	6 (15.0)	2 (6.3)	6 (18.2)	
High school	7 (17.5)	7 (21.9)	5 (15.2)	
Intermediate	3 (7.5)	6 (18.8)	9 (27.3)	
Graduate	12 (30.0)	7 (21.9)	12 (36.4)	
Parental consanguinity	0	0	0	NA
Surgical history	2 (5.0)	0	1 (3.0)	0.45
Medication on a regular basis	9 (22.5)	7 (21.9)	5 (15.2)	0.70
Exercise/yoga/meditation	3 (7.5)	2 (6.3)	1 (3.0)	0.71
Industrial locality	5 (12.5)	10 (31.3)	3 (9.1)	0.04
Tea/coffee	39 (97.5)	30 (93.8)	33 (100.0)	0.31
Smoking	6 (15.0)	13 (40.6)	15 (45.5)	0.01
Alcohol	6 (15.0)	8 (25.0)	11 (33.3)	0.18
Tobacco	3 (7.5)	8 (25.0)	5 (15.2)	0.12
BMI				
Underweight (<18.5 kg/m^2^)	8 (20.0)	1 (3.1)	4 (12.1)	0.24
Normal weight (18.5-24.9 kg/m^2^)	28 (70.0)	26 (81.3)	24 (72.7)	
Overweight (25.0-29.9 kg/m^2^)	2 (5.0)	4 (12.5)	3 (9.1)	
Obese (>30 kg/m^2^)	2 (5.0)	1 (3.1)	2 (6.1)	

## Discussion

The current study thoroughly analyzed the maternal and paternal demographic and lifestyle factors to assess their correlation with oocyte and embryo quality in IVF. Maternal factors such as tubal blockage and residence in an industrial locality were found to be significantly associated with the quality of oocytes. Although none of the female factors studied were associated with embryo quality, day 3 and day 5 embryo quality was significantly related to educational status of the male partners, smoking, and chewing tobacco. Day 5 embryo quality also associated with residence in an industrial locality of the male partners.

Anti-mullerian hormone (AMH) level has been reported to be substantially lower in women with tubal blockage than in women without tube obstruction [14]. This might explain the negative association of tubal blockage with oocyte quality observed in the present study as AMH is a useful marker of ovarian reserve in reproductive-aged women [15]. We observed in the present study that females with tubal blockage has significantly higher proportion of poor-quality oocytes. Also, the success rate of IVF can be affected by blocked fallopian tubes brought on by hydrosalpinx, a fluid accumulation within the fallopian tubes. Because of this, it might be challenging for embryos to implant because fluid in the fallopian tubes can interfere with the lining of the uterus [16]. Therefore, tubal blockage may indirectly affect the oocyte health.

The pool of primordial follicles governs the quantity and quality of a woman's reproductive life in the ovaries at various stages of development. The effects of environmental exposure on female fertility must be identified because occupational and environmental toxicant (ET) exposure is now unavoidable. Exposure to heavy metals, agricultural chemicals, tobacco smoke, and other chemicals used in the plastics, cosmetics, and sanitary product sectors, among other things, harm female fertility. Numerous investigations conducted in vivo, in vitro, and epidemiologically have shown that these ETs can alter folliculogenesis and decrease female fertility [17]. In line with these observations, in the present study, it was observed that women living in an industrial locality had poor-quality oocytes as compared to women living in non-industrial areas.

Studies have shown that paternal factors such as smoking, alcohol consumption, and chewing tobacco are all associated with poor pre-implantation embryo quality. A recent study showed a negative association of paternal smoking with the quality of pre-implantation embryos [18]. All semen parameters, including volume, density, concentration, and morphology related to reproductive success, can be negatively impacted by paternal smoking and high BMI. Smoking is known to exacerbate DNA deterioration and aneuploidies in sperm and to be related to or even the cause of congenital abnormalities [19]. Recent research demonstrates that sperm epigenetic information and DNA damage are passed on to the developing embryo [20,21]. We observed that embryo quality was negatively associated with paternal smoking. Couples in which the male partners smoked had higher prevalence of poor-quality day 3 and day 5 embryos than the non-smokers. The impact of paternal smoking on the pre-implantation embryos observed in the present study might be explained by the negative effects of smoking on the sperm quality, which may result in less optimum early development and consequently lower embryo quality.

Cigarette smoke has several toxic compounds having negative effects on male and female fertility. Smoking has been shown to affect endometrial thickness on the day of embryo transfer negatively. This may explain the harmful effects of tobacco smoke on implantation and pregnancy rates during ART [22]. A decline in sperm quality and, to a lesser extent, oligo-asthenozoospermia (or azoospermia) are highly related to Indian males with infertility evaluation. The detrimental effects of tobacco chewing on sperm quality should be discussed with infertile males opting for IVF [23].

It has been shown that tobacco chewers have a higher incidence of teratozoospermia. However, failures in many cases due to delayed fertilization, abnormal cleavage rates, and spontaneous abortions are known, [24] which could be, a priori, due to defective sperm characteristics, those impeding the normal event of fertilization [25,26]. This might explain the negative association of chewing tobacco with the embryo quality observed in our study.

Access to health care has been proven to rely independently on educational level [27]. The World Health Organization (WHO) claims that low education causes an increase in maternal and infant mortality and morbidity in developing nations [28]. Education generally has a favorable effect on a variety of health problems. Lifestyles and social views are influenced by education. Mixed views are available on the association of women’s educational status with the success of IVF [29]. However, no studies till date have evaluated the effect of paternal educational status on IVF outcomes. In the present study, we observed that paternal education was significantly associated with embryo quality. This might be due high awareness of the male partners about the precautions and the course of clinical management of the female partner during the IVF procedure.

We did not find a significant association of female factors with the embryo quality. This is in line with other studies highlighting no association of maternal demographic and lifestyle factors with embryo quality and IVF outcomes [30].

Although there is no consensus on the association of paternal lifestyle habits with embryo quality and IVF outcomes, couples planning to conceive either naturally or through ART should be advised to follow a healthy lifestyle.

Although the present study comprehensively analyzed several lifestyle factors of the couples opting for IVF, the findings are limited in terms of generalization due to the small sample size. Studies with larger sample sizes should be conducted to further substantiate the findings of the present study.

## Conclusions

Paternal lifestyle habits, such as smoking and chewing tobacco, and demographic characteristics, such as low education and proximity to an industrial area, were all related to poor embryo quality. Maternal factors such as tubal blockage and residence in the industrial locality were found to be negatively associated with the quality of oocytes. Therefore, paternal and maternal factors may influence embryo quality and IVF outcomes.
